# Evaluating different combination methods to analyse ultrasound and shear wave elastography images automatically through discriminative convolutional neural network in breast cancer imaging

**DOI:** 10.1007/s11548-022-02737-6

**Published:** 2022-08-26

**Authors:** Rudolf Hoffmann, Christoph Reich, Katrin Skerl

**Affiliations:** 1 Faculty Mechanical and Medical Engineering, Furtwangen University of Applied Science, Villingen-Schwenningen, Germany; 2 Faculty Informatik, Institute for Data Science, Cloud Computing and IT Security (IDACUS), Furtwangen University of Applied Science, Furtwangen, Germany; 3 Faculty Health, Safety, Society, Institute of Technical Medicine (ITeM), Furtwangen University of Applied Science, Furtwangen, Germany

**Keywords:** Machine learning, Deep learning, Discriminative convolutional neural network, Ultrasound, Shear wave elastography, Medical imaging

## Abstract

**Purpose:**

Ultrasound (US) and Shear Wave Elastography (SWE) imaging are non-invasive methods used for breast lesion characterization. While US and SWE images provide both morphological information, SWE visualizes in addition the elasticity of tissue. In this study a Discriminative Convolutional Neural Network (DCNN) model is applied to US and SWE images and their combination to classify the breast lesions into malignant or benign cases. Furthermore, it is identified whether analysing only the region of the elastogram or including the surrounding B-mode image gives a superior performance.

**Methods:**

The dataset used in this study consists of 746 images obtained from 207 patients comprising 486 malignant and 260 benign breast lesions. From each image the US and SWE image was extracted, once including only the region of the elastogram and once including also the surrounding B-mode image. These four datasets were applied individually to a DCNN to determine their predictive capability. Each the best US and SWE dataset were used to examine different combination methods with DCNN. The results were compared to the manual assessment by an expert radiologist.

**Results:**

The combination of US and SWE images with the surrounding B-mode image using two ensembled DCNN models achieved best results with an accuracy of 93.53 %, sensitivity of 94.42 %, specificity of 90.75 % and area under the curve (AUC) of 96.55 %.

**Conclusion:**

This study showed that using the whole US and SWE images through DCNN was superior to methods, in which only the region of elastogram was used. Combining breast cancer US and SWE images with two ensembled DCNN models in parallel improved the results. The accuracy, sensitivity and AUC of the best combination method were significantly superior to the results of using a single dataset through DCNN and to the results of the expert radiologist.

## Introduction

Breast cancer is one of the biggest health threats and the second leading cause of cancer death for women. An early diagnosis of breast cancer increases the survival rates [1]. There are several methods to diagnose breast cancer including Mammography, Magnetic Resonance Imaging and Ultrasound (US) imaging. The capability of US to detect lesions early is an important feature for its significance in breast cancer prognosis. It is a first-line imaging tool for breast lesion characterization [2]. In recent years, a new technique to measure the stiffness of the tissue was introduced and is in clinical use since 2009. This technique, called Shear Wave Elastography (SWE), is an US imaging modality that visualizes the elasticity of the tissue as a colour map superimposed on a greyscale US image, also called elastogram [3]. Morphological features can be extracted from US images. These features provide important structure and shape knowledge [1]. Since malignant lesions are usually stiffer than benign ones, elasticity of tissue can be helpful for identification [4]. Although SWE provides both morphological and elasticity information, it alone cannot improve the breast cancer diagnosis, but in combination with US [5].

A retrospective study regarding the recognition of breast cancer was conducted, in which two expert radiologists analysed US and SWE images to make a diagnosis [6]. In this study the diagnostic performance was evaluated and compared using solely US and SWE images as well as the combination of both images. This study indicated that using SWE images alone for diagnosis does not provide sufficient accuracy. Viewing SWE with US images at the same time, a radiologist may effectively improve diagnostic performance [6]. Another study showed that using US images with SWE images and a deep learning-based computer-assisted diagnosis software, that provides an assessment on malignancy of breast masses, improves the specificity and AUC without loosing sensitivity [7]. The additional information helps the radiologist evaluating breast masses, at which the complementary tools are more helpful for less experienced radiologists. US imaging requires professional competency and practical experience, since it is a handheld imaging modality and not yet as standardized as other imaging modalities [8]. An automated recognition of anatomical structures can minimize the dependency of examiner and optimize the analysis of large image databases [9]. Hence, applying novel approaches, such as machine learning algorithms, in clinical routine might help to further improve the diagnosis of breast cancer. In recent years, Discriminative Convolutional Neural Network (DCNN) has achieved great success in many problems of machine learning and computer vision due to its excellent performance in image recognition tasks. DCNN can automatically extract features or information from images and perform a classification based on quantitative evaluation of the extracted information [2]. There are several works, in which models were developed that automatically extract features from breast cancer US or SWE images and perform classification based on these features [1, 2, 10]. These works show that using DCNN gives a similar diagnostic performance than the manual image evaluation while being much faster than the manual evaluation. In [11] thyroid nodules are classified as benign or malignant using the combination of US and SWE images based on Convolutional Neural Network. The combination of these images improves the classification of thyroid nodules compared to just using a single data source method. These results are very promising but to the best of our knowledge, this methodology was not yet applied to breast cancer US and SWE images. Furthermore, only the region of elastogram was used through DCNN, which actually means that image information was dropped that may contain relevant medical information.

In this study we aim to evaluate different combination methods using breast cancer US and SWE images through DCNN. Furthermore, we aim to identify whether analysing only the region of the elastogram or including the surrounding B-mode image gives a superior performance. The performance of the algorithm shall be compared to the manual image evaluation by an expert radiologist with more than 20-year experience in breast cancer imaging.

## Material and methods

### Dataset

**Fig. 1:**
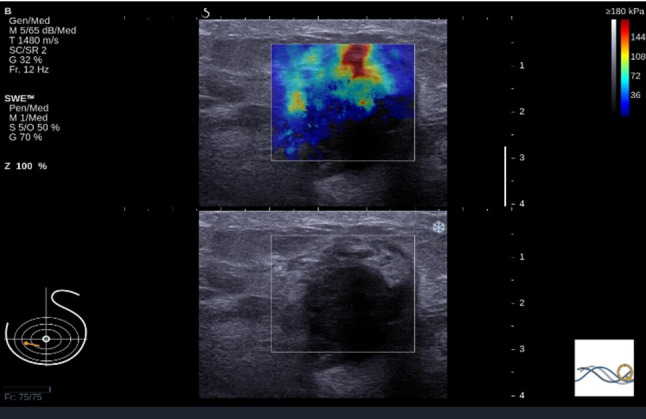
Example of an image from the original dataset

**Fig. 2:**
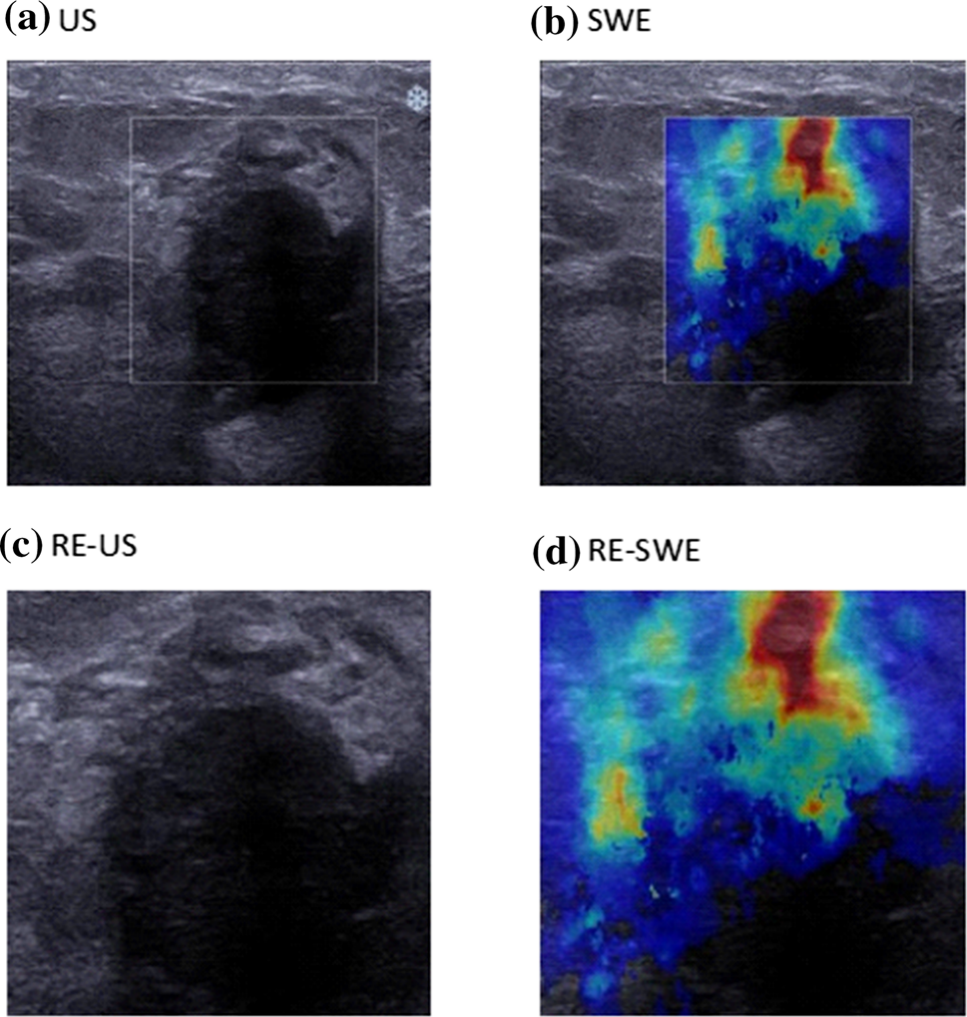
We cropped the images from the original dataset into: a) the entire B-mode image (US), b) the SWE image including the surrounding B-mode image (SWE), c) the B-mode image only in the region of the elastogram (RE-US) and d) the SWE image only in the region of the elastogram (RE-SWE)

The original dataset used in this study comprised 746 JPG images obtained from 207 patients comprising 486 malignant and 260 benign breast lesions obtained in a previous study [3]. All images were acquired with the Aixplorer ultrasound imaging system. The overall image captured by this system contains the grey-scaled US and coloured SWE image (see Fig. 1). Both images have the same dimensions. The difference between these images is the colour map in the SWE image that is superimposed on the pure US image in the region of elastogram selected by a radiologist. Furthermore, the surrounding of the US and SWE images contains labels that describes the properties used for the imaging, such as scale or frequency. For image classification with DCNN, this surrounding is redundant and not necessary. Using this surrounding causes vastly much more training time and resources for the model building and can be perturbing. In this work, the images were copied and cropped. From each image the US and the SWE image was extracted, once including only the region of the elastogram and once including also the surrounding B-mode image as shown in Fig. 2. These four datasets were resized to a squared dimension with a height and width of 224 pixels.

### Preprocessing the dataset

Each image from the original dataset was cropped into four images (see Fig. 2). The resulted images depict different dimensions, however, supervised machine learning models expect the same dimensions for input data. All images were resized to the shape (224, 224, 3), in order to be able to use the power of pre-trained Convolutional Neural Networks such as VGG16, if computational power is not available to train a model from scratch. This shape represents a 224x224 image with 3 channels. The resizing was performed by scaling the dimensions to the needed value. For the resizing, bilinear interpolation with anti-aliasing was used. For this, the image was blurred before down-sizing. Another possible strategy was cropping or padding the images, but in this work, this strategy was not suitable, because valuable information for the classification may get lost. Since the cropped images are not equal in their width and height, the morphology changes during resizing. In this work, the optimization of resizing was not performed.

### Creating a DCNN model

#### Architecture and hyperparameters

For the image classification, DCNN was selected, because it has achieved great success in image object detection and is thus suitable for this image recognition task. DCNN can automatically extract features or information from images and perform a classification based on quantitative evaluation of the extracted information [2].

In the first step, a DCNN model with four convolutional layers (Conv2D) was developed. The architecture is shown in Table 1. The DCNN model was created using TensorFlow and Keras. The used activation function for the hidden layers was Rectified Linear Unit (ReLU), while Sigmoid was used for the classification layer to constrain output between zero and one. The two-dimensional Max Pooling layer (MaxPool2D) was used for down-sampling to reduce the complexity for further layers. The Batch Normalization layer (BatchNorm) was used to normalize the values of the hidden units. This achieves a steadier network and reduces training time. The Dropout layer (Dropout) was used to prevent overfitting by dropping arbitrary hidden units during training time [12]. To perform classification, the extracted feature maps were flattened to a one-dimensional vector (Flatten), fully connected with 1024 units and then passed to a layer with only one unit (Dense) that represents the classifier. The built model was compiled and fitted with following parameters:

- optimizer: Adam with a learning rate of 0.0001
- loss: binary_crossentropy
- metric: BinaryAccuracy and AUC
- batch_size: 32
- epochs: 100

**Table 1:** Architecture of the DCNN model representing the layer and used filter or unit, kernel size (KS), padding (P), stride (S) and activation function (Act-Fct)

Layer	Filter/Unit	ks	P	S	Act-Fct
Conv2D	64	(11,11)	Same	4	ReLu
MaxPool2D	–	(3,3)	–	2	–
BatchNorm	–	–	–	–	–
Conv2D	128	(5,5)	Same	1	ReLu
MaxPool2D	–	(3,3)	–	2	–
BatchNorm	–	–	–	–	–
Conv2D	256	(3,3)	Same	1	ReLu
Conv2D	256	(3,3)	Same	1	ReLu
MaxPool2D	–	(3,3)	–	2	–
Dropout	–	–	–	–	–
Flatten	–	–	–	–	–
Dense	1024	–	–	–	ReLu
Dense	1	–	–	–	Sigmoid

#### Examining the predictive capability of the different datasets

The images from the original dataset were cropped into four images as described in Sect. 2.1. Most studies, such as [1] or [11], are focusing only on the region of elastogram of the US and SWE images. However, we compared the images that represent only the region of elastogram with the images that represent also the surrounding B-mode image. All datasets were used to build the DCNN model described in Sect. 2.3.1. In total, four different DCNN models were created and their predictive capability through DCNN was compared.

#### Combining US and SWE images

**Fig. 3:**
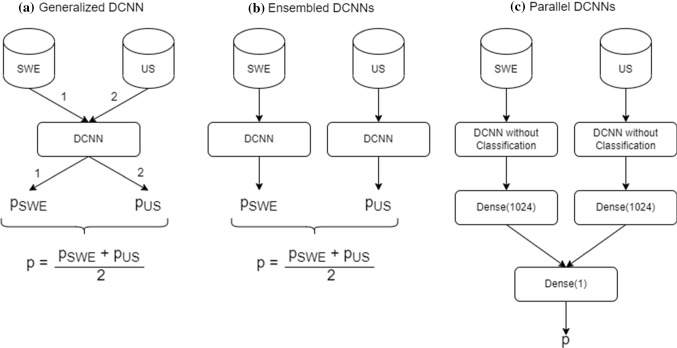
Networks of different Combination Methods. **a** Generalized DCNN - One DCNN model is built using US and SWE images. For the classification, US and SWE images are fed to the model and the outcome probability is averaged. **b** Ensembled DCNNs - Two identical DCNN models are built. The first model uses US images, the second model uses SWE images. For the classification, the outcome probability is averaged. **c** Parallel DCNNs - Two identical DCNN models without a classification layer are fed with US and SWE images. The features from both datasets are extracted, combined and passed to a classification layer

To combine the images, three different methods were examined. In the first method, US and SWE images were individually used to build the single DCNN model as shown in Fig. 3a Generalized DCNN. The decision was made by feeding this model with both images and averaging the outcome probability of both images. The idea of this method was to achieve a generalized model that uses the combination of US and SWE images for breast cancer detection, but can also work with US and SWE images alone.

The second method (see Fig. 3b Ensembled DCNNs) used ensemble learning with two identical DCNNs to combine the datasets. One DCNN was built to work with US images and the other DCNN was built to work with SWE images. The outcome probability was averaged for decision making. The disadvantage over the first method is, that two models have to be built, however, working with a single dataset is still possible.

In the third method, US and SWE images were fed to two identical DCNNs without a classification layer in parallel. In this method, the morphological and elasticity information from the US and SWE images was extracted simultaneously and these extracted features were combined and passed to a classification layer for the decision making (see Fig. 3c Parallel DCNNs).

#### Evaluation

For modelling, the entire dataset was split into three parts as shown in Table 2. In order to evaluate the model during the training process, the validation data was used. This way, the model was tuned to give a good prediction for the validation data. To test the overall performance of the model, the test/evaluation data, that was unknown for the model so far, was used. Every model was created and evaluated 20 times. The results in this work represented the mean and standard deviation of these 20 models.

#### Statistical significance of the model

The results achieved with the combination of US and SWE images were tested for statistical significance. Therefore, the results achieved with the use of a single dataset were considered as null hypothesis H0 and the results with the combinations of US and SWE images as alternative hypothesis H1. Furthermore, it was tested, if the examined approach was statistically significant compared to the results achieved by the expert radiologist in [6]. These results and thus the null hypotheses H0 were 90.10 % for accuracy, 92.60 % for sensitivity, 86.40 % for specificity and 91.30 % for area under the receiver operating characteristic curve (AUC). For testing statistical significance, the Z-score was used. Based on the Z-score, the p-value was calculated. Values of p < 0.05 are considered as statistically significant.

1 $$\begin{aligned} Z=\frac{x-\mu }{\sigma } \end{aligned}$$

To calculate the Z-score, the value of the null hypothesis was used for x, while the mean and the standard deviation from the results in this work were used for $\upmu$ and $\upsigma$. The p-value was calculated using a web-based calculator on the website of “Social Science Statistics” [13].

## Results

### Comparing the datasets

The first four rows of Table 3 summarize the results of using single datasets through DCNN. It indicates that SWE achieved with all metrics the best results, although it had to be scaled down more than RE-SWE, so more information got lost. However, analysing the SWE dataset could not outperform the results achieved by the expert radiologist from [6]. US achieved inferior results to SWE, but superior results to RE-SWE. RE-US achieved worst results of all datasets. However, the difference between the results achieved by the different datasets is not statistically significant.

**Table 2:** Splitting the dataset into training, validation and test/evaluation data

Part	Ratio (%)	Samples for method generalized model	Samples for other methods
Training	70	1044	522
Validation	15	224	112
Test/Evaluation	15	112	112

The number of training and validation samples for the method Generalized DCNN was twice the size of the other methods, because two datasets were used to fit the model. However, the number of test/evaluation samples was of same size, because the outcome probability of both datasets was averaged for the prediction

Evaluating the region of elastogram of the SWE and US images only gave an inferior performance. Thus, in the following only the images including the surrounding B-mode image (SWE and US) were analysed further.

### Combining US and SWE

Table 3 compares the results of the different combination methods using US and SWE images with the results of using RE-US, RE-SWE, US and SWE images alone and the expert radiologist from [6]. Combination method Generalized DCNN had a statistically significant higher sensitivity and AUC, but an inferior specificity than using US and SWE alone. No significant difference was observed in accuracy. Although the AUC was statistical significantly higher than the manual evaluation, all other metrics gave inferior results. Combination method Ensembled DCNNs achieved a statistically significant higher accuracy, sensitivity and AUC than using the US and SWE images alone and than the expert radiologist. Combination method Parallel DCNNs achieved better results in all metrics than US and SWE images alone. However, the results were not statistically significant superior. Only the specificity was statistically significant superior to the result of the expert radiologist. The best diagnostic accuracy, sensitivity and AUC were achieved using method Ensembled DCNNs. However, if specificity was more important, method Parallel DCNNs gave the best performance.

**Table 3:** Comparing the results of different combination methods using US and SWE images with the results of using RE-US, RE-SWE, US and SWE images alone and the expert radiologist from [6]

Method	Acc (%)	Sn (%)	Sp (%)	AUC (%)
	$\upmu$, $\upsigma$	$\upmu$, $\upsigma$	$\upmu$, $\upsigma$	$\upmu$, $\upsigma$
Manual evaluation	90.10	92.60	86.40	91.30
RE-SWE	86.07, 4.73	76.29, 13.90	90.80, 6.87	83.55, 6.43
RE-US	82.37, 5.65	72.15, 11.92	88.16, 8.11	80.16, 5.75
SWE	89.82, 3.82	80.17, 14.02	94.79, 3.54	87.48, 6.42
US	88.17, 2.97	80.10, 6.27	92.53, 3.97	86.32, 3.21
Generalized DCNN	88.45, 1.80	87.97, 1.62	79.71, 3.00	**96.56, 2.48**
Ensembled DCNNs	**93.53, 1.32**	**94.42, 0.92**	90.75, 3.55	**96.55, 2.21**
Parallel DCNNs	93.31, 2.85	89.09, 5.41	**95.52, 2.97**	92.30, 3.20

Results that are statistically significant superior to the results of a single dataset are indicated with an underline and to the results of the expert radiologist are indicated bold

## Discussion of the results

In this work, a DCNN model was built from scratch that was fed with US and SWE images and classified them into malignant or benign cases. The images from the original dataset were cropped into four images: US, SWE, RE-US and RE-SWE. After the cropping, the images had various dimensions, but in most cases, the width was greater than the height. The bilinear interpolation was used to resize the images to square shapes. Cropping the images or padding them with zeros to appropriate dimensions was not done, since valuable information may get lost or too many resources would be required. However, the resizing to square dimensions led to morphological changes. This might have biased the results. Resizing the images to unified dimensions, but with a ratio, in which the width is greater than the height, would probably improve the results.

Comparing all images indicated that US and SWE achieved superior results to RE-US and RE-SWE. This led to the assumption that valuable information beyond the region of elastogram is available. The images had to be down-scaled. The more the images are down-scaled, the more information gets lost. In order to increase performance regarding to the needed resources and training time, the images were resized to a shape of (224, 224, 3). Using the dataset with the greater shape would further improve the results.

For the combination of the images, three different methods were used. In combination method Generalized DCNN, a DCNN model was built with single US and SWE images. For the prediction, this model used both US and SWE images and averaged their outcome. This combination method achieved worst results compared to the other combination methods. Compared to the results of using single US and SWE images through DCNN, the accuracy was quite similar, the specificity decreased, but the sensitivity and AUC increased. Probably the weights of the DCNN model were not able to determine good values, because two different types of images were used for the model fitting. Combination method Ensembled DCNNs used two identical DCNNs to combine the datasets. One DCNN was fit with US and the other DCNN was fit with SWE images. The outcome probability of both models were averaged for the decision making. This way, the problem from method (finding good weights) was vanished, because one model determined the weights for US images, while the other model determined the weights for SWE images. The results of combination method Ensembled DCNNs were, except of specificity, statistically significant compared to the results of the single dataset usage and the expert radiologist. Method Generalized DCNN and method Ensembled DCNNs have the advantage, that they do not necessarily require US and SWE images simultaneously, but can also make a prediction with one of them. Method Generalized DCNN has the advantage that only one DCNN model has to be built in contrast to method Ensembled DCNNs, but method Ensembled DCNNs achieved significantly better results. Combination method Parallel DCNNs used two DCNNs without a classification layer in parallel to extract the features from US and SWE images that were combined and used for the classification. The results of this method yielded also an improvement, however only the specificity was statistically significant. In addition, combination method Parallel DCNNs can only work with both US and SWE images simultaneously and is not as general as combination method Generalized DCNN and method Ensembled DCNNs.

In [11] three different combination methods of RE-US and RE-SWE images were used to classify thyroid nodules in malignant or benign cases. The worst combination method in that study mixed the two datasets to one common dataset. This method is similar to our method Generalized DCNN. However, we extended this method by using the outcome probability of both datasets for the prediction. Another method examined in that study was the fusion of RE-US and RE-SWE images to a six channel tensor. This method was not examined in our study, because the results were similar to the other method and significantly worse than the best method. The best method of that study is identical to our method Parallel DCNNs and achieved also results that are statistically significant better than the results of the manual evaluation. As we see, the combination of US and SWE images has the potential to improve the results in both thyroid nodule imaging as well as breast lesion imaging. Hence, this method can be extended to further areas of application on organic site. Our best combination method Ensembled DCNNs was not used in that study. Possibly, using ensemble learning with US and SWE images instead of RE-US and RE-SWE images of thyroid nodules could also improve the results of that study.

Our study has several limitations. The size of the surrounding region as well as the position of the elastogram differs and may affect the results. Besides, the freeze symbol in the US images and the frame of the elastogram in the US and SWE images are visible. This additional information in the images may be perturbing for the DCNN model that automatically extracts features from the images. Furthermore, the dataset used in this study is the same part of the dataset used by the expert radiologist in [6] but not identical to it. Comparing the results of the manual evaluation and our algorithm using the identical dataset would be more meaningful.

## Conclusion

In conclusion, including the surrounding B-mode image into the analysis gave a higher predictive capability than analysing the region of the elastogram only, both for SWE and US images. The combination of US and SWE images using two ensembled DCNN models yields better results than using a single dataset through a DCNN model. The presented combination method using DCNN has a high predictive capability to differentiate breast lesions into malignant or benign cases. The accuracy, sensitivity and AUC are statistically significant superior to the results of using a single dataset through DCNN and to the results of the manual evaluation. Thus, the introduced model gives a superior outcome compared to the clinical standards and has the potential to improve breast cancer detection using US and SWE images.
